# New tests, new tools: mobile and connected technologies in advancing psychiatric diagnosis

**DOI:** 10.1038/s41746-017-0006-0

**Published:** 2018-02-26

**Authors:** Laura Weiss Roberts, Steven Chan, John Torous

**Affiliations:** 1 Department of Psychiatry and Behavioral Sciences, Stanford University School of Medicine, Stanford, CA USA; 2 Department of Psychiatry and Division of Hospital Medicine, Clinical Informatics, University of California San Francisco, San Francisco, CA USA; 3 Department of Psychiatry and Division of Clinical Informatics, Beth Israel Deaconess Medical Center, Harvard Medical School, Boston, MA USA

**Keywords:** Diagnostic markers, Diagnosis

## Abstract

Mental health is an area of growing interest in the digital health space. Mobile and connected technologies offer new tools that can potentially aid in both the diagnostic and interventional aspects of psychiatric care. To understand the potential of these digital tools in psychiatry, this paper offers an overview of the development, current research, clinical use cases, and next steps necessary to realize the potential of digital health in mental health. Focusing on smartphones’ and wearable sensors’ ability to advance clinical data collection via multiple domains: self-report, behavioral, and physiological, we explore opportunities and challenges in translating these technologies into clinical care tools that can advance how we understand and approach mental illness.

## Introduction

Medical informatics, computer science, and engineering innovations have heralded a new era in clinical medicine. Some advances, such as the evolution of electronic medical records to document, communicate, and support clinical decision-making, are quickly becoming commonplace.^1^ Yet many of the potential uses and advantages of new technologies for healthcare remain nascent.^2^ In light of the prevalence of mental disorders,^3^ the lack of adequate services,^4^ and numerous barriers to optimal clinical care,^5^ there is a now sense of anticipation and hope that new technologies will be valuable in fostering mental health.^6^ New e-tools are being rapidly developed, and will allow for collection and analysis of data derived from self-reports, monitoring of behavioral patterns, and physiological sensing. Data of each type may have important and beneficial applications. And yet, by triangulating these different forms of e-data that signal clinically salient phenomena, it is possible to create novel diagnostic tests for psychiatry. These new diagnostic tests, we suggest, may be adopted widely and used by practitioners across the health professions, including in primary care and community-based settings. Moreover, the tests very importantly may help improve our capacity to identify conditions early and to intervene in ways that support positive mental health outcomes for individuals and populations.

## Development of e-tools in psychiatry

Several factors at the national policy, patient, and technology level together have created an opportune time for psychiatry to begin to realize the potential of mobile and connected technologies. National policies now favor the use of technologies with recent implementation of the Mental Health Parity and Addiction Equity Act of 2015 and the 2016 Centers for Medicare and Medicaid Services announcement of financial incentives for behavioral health information technologies. Enthusiastic patient and consumer use of mobile devices, a high prevalence of connected mobile devices with standardized operating systems, and the availability of high-speed wireless internet are factors making it easier for patients and providers to access new interactive tools to quantitatively measure oneself.

These factors have culminated in patient and provider interest in monitoring mental health symptoms. The ability of consumer technologies like smartphones to now capture this wealth of behavioral and physiological data that previously was only possible with customized actigraphy devices presents a sea of “big data” for psychiatry that has never before been available. For example, it is possible to now collect over one million data points per patient per day via smartphone apps.^7^ There is currently a perfect alignment of health policies, patient and consumer technology ownership and interest, and the sensor and computing power of new mobile technologies, thus translating to new opportunities for psychiatry.

Excitement surrounding such opportunities for new technologies should be tempered by the observation that very few e-tools have been well studied or validated, and overall we have little experience with their impact when implemented on a broad scale.^8^ Interest in mobile and connected technology in the arena of mental health is dramatically expanding,^9^ while much of this growth is occurring in the private sector—without being informed by evidence, expertise, or ethics of the profession of medicine.^10^ As the practice of psychiatry will certainly be transformed by business-driven innovation in mobile and connected technologies, it is essential that psychiatrists become aware of how technology will affect the health of our patients in the future. Furthermore, it should be possible to explore the role of technology in clinical care, while ensuring that its use remains both practical and actionable.

## e-Data and diagnostics in psychiatry

The Diagnostic and Statistical Manual-5 is the guiding document for the diagnosis of mental disorders throughout the world. The DSM-5 introduced experimental crosscutting symptom measures based on psychometric assessments—essentially questionnaires that quantify symptoms. These measures have limitations, however, such as inter-rater reliability, cultural and linguistic discrimination, and subjectivity in interpretations and usage of such questions. Alternatively, the National Institute of Mental Health Research Domain Criteria (RDoC) criteria^11^ creates another approach that may complement the more phenomenological diagnostic framework of DSM; RDoC focuses on domains such as negative valence systems, positive valence systems, cognitive systems, systems for social processes, and arousal/regulatory systems; RDoC is a novel framework that has value in translational research, but its use and utility in clinical practice remain untapped. Research on genetic, molecular, neuroimaging, and psychosocial phenotypic biomarkers for diagnosis has made progress as well, although not currently used in everyday clinical practice.

Mobile technology has the versatility to engage with DSM, RDoC, and other scientific frameworks, separately and in combination. This remarkable capability represents a new lens through which we may better observe and understand patterns associated with mental disorders. Through real-time passive and active data collection, smartphones and connected technologies can potentially unite both the biological and behavioral sides of psychiatry^12^ and provide a novel digital phenotype that may guide or support diagnostic decision making.^13^ We suggest that mobile and connected technologies can provide diagnostic data within three units of analysis: self-reported symptoms, behavioral patterns, and physiological parameters.

### Self-reported data

Diagnoses in psychiatry in routine clinical care are largely based on the retrospective reports of patients (“How have you been sleeping over the past few weeks?”, “How is your mood most days?”, “When did you notice that you had no appetite and started losing weight?”). Retrospective recollection of symptoms can be even more difficult given that many psychiatric disorders—for instance, schizophrenia, major depressive disorder, neurocognitive disorders such as dementia—are associated with cognitive or memory deficits that may make accurate recalling of past symptoms difficult.

Mobile and connected technologies can offer ways to capture self-reported data in real time, pushing the process of data collection out of the office and into the ecology of the everyday world of patients. A recent Cochrane review on self-administered health surveys on apps noted that apps may not adversely affect data equivalence as long as the intended clinical use, frequency, and setting of the survey remains unchanged from the setting that the survey was validated in.^14^ Yet the potential of mobile devices to capture mood is that they offer a new setting outside of the clinic and potential to offer more frequent assessments, which underscores the need for new scales.

Currently, none of the often-used clinical questionnaires have been designed and validated for use on a mobile device or use outside of the clinic, with few randomized clinical trials for electronic self-monitoring in moods.^15,16^ Early evidence has demonstrated that even the simple translation of a basic scale—like the PHQ-9 onto a smartphone—can produce very different data than when collected face to face in a clinic.^17^ With a better understanding of the validity of self-reported symptoms surveys via mobile devices, the potential of adaptive surveys that can customize assessment to each patient becomes possible. Such computerized-adaptive testing intelligently asks questions to diagnose,^18,19^ but the adoption of such systems is hampered by the limitations in trials so far—such as the lack of validation against existing scales, lack of test-retest reliability, and commercial licensing.^20^

These self-reported symptoms may potentially be more sensitive in diagnosing psychiatric illness and monitoring recovery^21^ as compared to classical clinical-based self-reported outcome measures. Thus, research efforts that are focused on validating new scales for psychiatric symptoms collected synchronously offer interesting potential to realize a new window into these illnesses. There is a need to study the potential of this new stream of self-reported data as a new diagnostic instrument for psychiatry.

### Monitoring of behavioral patterns

Psychiatric disorders are complex, and nearly all involve some degree of behavioral dysregulation or abnormality. Smartphones and connected devices—like smartwatches—have become inadvertent monitors of behavior. These technologies often monitor our location, acceleration, social activities—through patterns in phone calls, text messages, and social media posts—and voice features passively in order to keep us connected. This form of passive data may be diagnostic of illness: a patient with bipolar disorder who enters a manic phase demonstrates more phone activity evidenced from a combination of increased GPS position changes, erratic accelerometer movements, and increased social activity. However, early research on passive data and bipolar disorder has shown that these relationship are not simple, with passive data not directly matching clinical symptoms.^22^ These complexities of passive data have also been supported by recent studies on depressive symptomatology.^23^ In many ways the situation is similar to EEGs which can offer millions of data points about a patient’s neural activity although such data may still have limited clinical utility. There is a need for clinically focused research that seeks to validate various passive data streams and determine whether there is a useful clinical signal or noise here.

Another behavioral marker of high interest is voice data. Given the potential of the pitch, tempo, and loudness of voice to serve as a biomarker of many psychiatric illnesses and states, such as depression, anxiety, and even suicidality,^24^ it will be important to validate real-time voice data as diagnostic markers. Automated speech analysis of patient transcripts, combined with machine learning and natural language processing, can measure subtle mental state changes in psychosis.^25^ This type of analysis is already being used in marketing, customer service call centers, and business intelligence services—although it is not commonly utilized in day-to-day clinical care.

There are numerous other potential biomarkers that the phone collects, which could soon serve as diagnostic tests. But for now, passive data for psychiatry is poorly understood and not yet ready for clinical use. With the high potential for objective markers of illness, there is a need for diagnostic research to offer more reliable guidance for this promising area to come to fruition.

### Physiological sensing

While self-reported symptoms and behavioral observations are important in the diagnosis of psychiatric illness, directly measured physiological data (e.g., heart rate, sleep quality, skin conductance) are less often considered less often. There is nevertheless a large literature on actigraphy in psychiatry, with promising results such as heart rate variability data as a marker of decompensation in schizophrenia.^26^ Collecting real-time physiological data has been impractical as such devices have traditionally been bulky, large, and expensive. These feasibility challenges have limited the use of physiological sensing both in the psychiatric patient’s everyday life and in the psychiatrist’s everyday practice.

With smartphones and fitness tracking devices readily available, it is now easier to collect this new stream of biologically significant data. Early studies have shown that patients with serious mental illnesses are adherent to and even enjoy using fitness trackers to collect this type of data.^27^ But there is a need for more research on the validity of data collected with consumer technologies, and on how information collected on these platforms correlates with illness. Physiological data, we suggest, may well prove to be important diagnostic measures in clinical psychiatry practice of the future.

## Triangulating e-data to create useful psychiatric tests?

While mobile and connected technologies can likely capture valuable data for psychiatric diagnosis at the self-report, behavioral, and physiological levels, it is possible that no one single test will be diagnostic. Rather, it may be that a combination of various real-time mobile collected data streams must be combined to create personalized diagnostic information. The methods to realize these personalized diagnostics are also an area of active research^28^ and reflect the growing importance of data science in psychiatry.^12^ One potential reason for why psychiatry has not yet realized the potential of mobile and connected data is that most research efforts to date have focused on individual data streams, perhaps neglecting the contribution of several small disparate signals.

Before psychiatric practice can embrace these uses of e-data, it will be important to overcome the pervasive challenges associated with the lack of standardization and medical-grade calibration of device measurements. Hundreds of smartphone devices all use different combinations of sensors, CPU, GPU, and operating system versions. “False positives” can misinterpret activity patterns. For instance, a patient’s phone sensing a drop in sent text messages may be falsely attributed to an increase in vegetative symptoms of depression. On the other hand, this decrease in messaging activity could also be attributable to the patient who is traveling overseas without text messaging service, the patient who is commencing a week of strenuous overnight work, the patient who forgets to charge their device, or the patient who becomes frustrated with a phone’s increasingly slower performance.

## Conclusion

In his book *The Structure of Scientific Revolutions*, Thomas Kuhn writes that “truth emerges more readily from error than from confusion”. Currently, the potential of mobile and connected technologies have led to much excitement, some confusion, as well as rising hope that e-tools can help improve the lives of people in need. In approaching these technologies from a systematic research perspective and creating a framework to evaluate their actual effectiveness and triangulating information drawn from multiple sources, we can begin to discover whether there is clinical value to in realizing this great potential.

As yet, we have not seen mobile and connected technologies fundamentally transform routine diagnosis of psychiatric disease or other aspects of clinical psychiatric care. And thus far, with limited exceptions, we have also not seen strong efforts at scientific and clinical investigation of the new tests and new tools that mobile technology make possible. Now is the time to increase this research and to have such inquiry be guided by the aim of fostering patient well-being. Developing new tests and new tools that allow for early diagnosis and intervention in the context of mental disorders can do much to address human suffering and to reduce the extraordinary burdens associated with these conditions in our world.
